# The Association Between Posting WeChat Moments and the Risk of Depressive Symptoms Among Middle-Aged and Older Chinese Adults: Prospective National Cohort Study

**DOI:** 10.2196/62730

**Published:** 2025-01-13

**Authors:** Wei Wang, Hui Wang, Xinru Hu, Qian Yu, Fangyi Chen, Xirui Qiu, Xiaoxiao Wang

**Affiliations:** 1 GCP Center, Affiliated Hospital of Nanjing University of Chinese Medicine (Jiangsu Province Hospital of Chinese Medicine), Nanjing, China; 2 Public Health Service Center of Nanjing Jiangbei New District, Nanjing, China; 3 Department of Public Health, School of Medicine and Holistic Integrative Medicine, Nanjing University of Chinese Medicine, Nanjing, China; 4 Department of Science and Technology, Affiliated Hospital of Nanjing University of Chinese Medicine (Jiangsu Province Hospital of Chinese Medicine), Nanjing, China; 5 Department of Respiratory and Critical Medicine, Affiliated Hospital of Nanjing University of Chinese Medicine (Jiangsu Province Hospital of Chinese Medicine), Nanjing, China; 6 Clinical Research Institute, Affiliated Hospital of Nanjing University of Chinese Medicine (Jiangsu Province Hospital of Chinese Medicine), 155 Hanzhong Road, Nanjing, 210029, China, 86 13770784000

**Keywords:** cohort study, depression, depressive symptoms, mental health, middle-aged adults, modified Poisson regression, older adults, WeChat

## Abstract

**Background:**

The association between social media usage and the risk of depressive symptoms has attracted increasing attention. WeChat is a popular social media software in China. The impact of using WeChat and posting WeChat moments on the risk of developing depressive symptoms among community-based middle-aged and older adults in China is unknown.

**Objective:**

The objective was to assess the association between using WeChat and posting WeChat moments and the risk of depressive symptoms among middle-aged and older adults in China.

**Methods:**

A prospective national cohort study was designed based on the data obtained from the fourth and fifth waves of the China Health and Retirement Longitudinal Study (CHARLS). The strength of association between using WeChat and posting WeChat moments and the risk of depressive symptoms was estimated by modified Poisson regressions. Depressive symptoms were determined using the 10-item Center for Epidemiologic Studies Depression Scale. Meanwhile, the heterogeneity of the associations was explored through multiple subgroup analyses. Moreover, multiple sensitivity analyses were performed to verify the robustness of the associations between the exposures and depressive symptoms.

**Results:**

A total of 9670 eligible participants were included in the cohort study, and the incidence rate of depressive symptoms was 19.08% (1845/9670, 95% CI 19.07%‐19.09%) from the fourth to fifth waves of the CHARLS. Using WeChat (adjusted relative risk [aRR] 0.691, 95% CI 0.582‐0.520) and posting WeChat moments (aRR 0.673, 95% CI 0.552‐0.821) reduced the risk of depressive symptoms among middle-aged and older Chinese adults. The association between the exposures and depressive symptoms was robust, proved through multiple sensitivity analyses (all *P*<.05). However, the associations were heterogeneous in certain subgroup catagories, such as solitude, duration of sleep at night, nap after lunch, physical activity, and having multiple chronic conditions.

**Conclusions:**

Using WeChat and especially posting WeChat moments can mitigate the risk of depressive symptoms among community-based middle-aged and older Chinese adults. However, there is likely a need for a longer follow-up period to explore the impact of the exposures on the risk of long-term depressive outcomes.

## Introduction

Depression is a common emotional disorder [1-4] resulting in limited psychosocial functioning and a decreased quality of life [5] and is also an independent risk factor of all-cause mortality and other chronic conditions [6-8]. A meta-analysis [3] reported that the prevalence of depressive symptoms among older adults in mainland China was 20% (95% CI 17.5%‐22.8%), and Chen et al [9] reported an unweighted incidence rate of depressive symptoms of 34.77% based on the China Health and Retirement Longitudinal Study (CHARLS). These findings highlight the need for an increased attention to depressive symptoms in China. Therefore, understanding modifiable factors associated with depressive symptoms is essential for developing effective public health strategies to prevent them. Multiple well-known factors associated with depressive symptoms include genetic factors, psychosocial stress, changes in the hypothalamic-pituitary-adrenal axis, inflammation, and others [5,10,11].

In terms of psychosocial factors, there has been a growing body of studies on the association or correlation between social media usage (SMU) and depression in recent years. Studies conducted by Lin et al [12], Primack et al [13] and Perlis et al [14] demonstrated that SMU, particularly on Facebook, TikTok (known as Douyin in China) and Snapchat, was associated with an increased risk of depression among US adults. However, Cotten et al [15] analyzed the data from 6 waves of the National Health and Aging Trends Study and suggested that SMU may not be related to depression in older adults. Based on the above studies, the association between SMU and depression could be attributed to problematic SMU [16,17], while moderate and reasonable use of social media may not cause harm to mental health.

WeChat (known as Weixin in China) is a popular social media software in China, which had 1.04 billion monthly active users according to Tencent [18]. The primary functions of WeChat include texting, voice and video calling, and browsing and posting moments that encompass both positive and negative events happening among friends or oneself, as well as facilitating financial management. From the perspective of these functionalities, WeChat distinguishes itself from platforms like Facebook or TikTok, which focus on interests and preferences that can foster addiction or problematic SMU.

It is hypothesized that using WeChat and posting WeChat moments would not result in an increased risk of depressive symptoms among community-based middle-aged and older Chinese adults, but rather potentially mitigate the risk of developing depressive symptoms. In fact, two cross-sectional studies using data from the fourth wave of the CHARLS and the fifth wave of the China Family Panel Study reported an association between WeChat usage and a decreased risk of depressive symptoms among middle-aged and older Chinese adults [19,20]. However, the two studies were unable to determine a temporal relationship between WeChat usage and depressive symptoms, thus precluding any causal inferences regarding the relationship between the observed exposures and depressive symptoms. Additionally, findings from the study conducted by Qu et al [19] indicated that participants who posted WeChat moments did not exhibit a decreased risk of depressive symptoms, which contradicted the findings of Zhang and Liang [20].

In response to these conflicting findings, a national, prospective cohort study was designed, leveraging data from both the fourth and fifth waves of the CHARLS. The aim of this study was to elucidate the relationship between the use of WeChat—with particular emphasis on posting WeChat moments—and the risk of depressive symptoms.

## Methods

### Study Design and Study Population

The study was characterized as a national, prospective cohort study, deriving its data from the fourth and fifth waves of the CHARLS. Initiated in 2011, the CHARLS represents a longitudinal national survey aimed at collecting extensive data pertinent to community-based middle-aged and older adults in China. The scope of the CHARLS encompasses high-quality, representative panel data covering a broad spectrum of topics. These topics include demographic variables, socioeconomic status, familial relationships, health status, health care usage, and others. More information about the CHARLS is accessible at their official website [21].

The intersection of the following 3 datasets from the CHARLS were compiled for the raw dataset: demographic backgrounds from the fourth wave, health status and functioning from the fourth wave, and the 10-item Center for Epidemiologic Studies Depression Scale (CESD-10) from the fifth wave. Participants from the raw dataset that (1) were aged <45 years, (2) were missing scores for the CESD-10 in the fourth wave, (3) had a CESD-10 score ≥10 in the fourth wave, or (4) were missing scores for the CESD-10 in the fifth wave were excluded.

### Assessment of Exposures

In the fourth wave of the CHARLS, participants were asked, “Do you use WeChat?” with possible responses being yes or no. Participants who answered yes were categorized as WeChat users; otherwise, they were categorized as non-WeChat users. Subsequently, the participants using WeChat were further asked, “Do you post WeChat moments?” with possible responses being yes or no. Participants who answered yes were categorized as WeChat moments users; otherwise, they were categorized as non-WeChat moments users.

Therefore, according to whether or not they used WeChat, the participants were stratified into 2 groups: WeChat users and non-WeChat users. Furthermore, according to whether or not the participant posted WeChat moments, they were stratified into 3 groups: non-WeChat users, non-WeChat moments users and WeChat moments users. In this study, we first focused on the comparison between the incidence of depressive symptoms among the WeChat users and non-WeChat users, then that among non-WeChat users, non-WeChat moments users and WeChat moments users.

### Assessment of the Outcome

The outcome of this study was depressive symptoms, which were measured using the CESD-10 scores from the fifth wave of the CHARLS as a dichotomous variable (yes or no). Each item of the CESD-10 was rated on a 4-point Likert scale ranging from 0 to 3, corresponding to the frequency of the symptoms (0=rarely or not at all; 1=some or a little of the time; 2=occasionally or a moderate amount of time; 3=most or all of the time). The aggregate score, which was the sum of the responses to all items, thus ranged from 0 to 30. A cumulative score of 10 or above was used as a threshold for identifying participants with depressive symptoms, whereas scores below this threshold indicated the absence of such symptoms. Additionally, for sensitivity analysis, alternative cutoff points of 11 and 12 on the CESD-10 were also employed to further ascertain the presence of depressive symptoms.

### Assessment of Covariates

The following characteristics obtained from the fourth wave of the CHARLS were considered as covariates: age, sex (male or female), race (Han or minority), faith (yes or no), type of community (village or city/town), marital status (married and cohabiting, married and separated, or others), education (illiterate, not finished primary school/home school/primary school, junior high school, or senior high school and above), smoking habits (currently, ever, or never), alcohol consumption (yes or no), duration of sleep at night (<6 hours, ≥6 hours to <8 hours, or ≥8 hours), nap after lunch (yes or no), and physical activity (none, mild, moderate, or vigorous). Additionally, the social activity score was a covariate [22], which was the frequency of 11 kinds of activities rated as never (score=0), not regularly (score=1), almost every week (score=2), or almost daily (score=3). These activities were assembled to a sum score based on the frequency level, and the total scores for social activities could range from 0-33 points.

The basic activities of daily living (BADL) score was also a covariate [23], which included items for dressing, bathing, feeding, moving from a bed to a chair, using the toilet, and maintaining continence. All items had 4 potential answers, including (1) do it without difficulty, (2) do it but with difficulty, (3) do it with difficulty and need help, or (4) cannot do it, which were rated as 0, 1, 2, or 3 points, respectively. BADL scores were the sum of points for all items. Another covariate was life satisfaction. This question had 5 alternative answers: completely satisfied, very satisfied, somewhat satisfied, not very satisfied, or not at all satisfied. The responses were then stratified into 3 categories: completely/very satisfied, somewhat satisfied, or not very/not at all satisfied. Last, the number of chronic conditions (5 categories: no chronic conditions, 1 chronic condition, 2 chronic conditions, 3 chronic conditions, and ≥4 chronic conditions) and the number of parts with body pain were covariates.

### Statistical Methods

Descriptive analyses and differential comparisons were conducted on the characteristics of the eligible participants. The quantitative variables with a normal distribution were described using mean and SD, and a 2-tailed *t* test or one-way ANOVA was used for comparing the difference between two groups or among three groups, respectively. Quantitative variables with skewed distributions were described using the median and IQR, and a Wilcoxon rank sum test or Kruskal-Wallis H test was used for the comparison. Disordered multinomial variables were described by the number of cases and percentage, and a *χ*^2^ test or Fisher exact test was used for the comparison. Ordinal multinomial variables were described by the frequency and percentage, and a Wilcoxon rank sum test or Kruskal-Wallis H test was used for comparison.

For all eligible participants, univariable modified Poisson regressions were used to analyze the association between using WeChat and posting moments with the risk of depressive symptoms. Next, variables with *P*<.05 from between-group comparisons, along with adjusted variables in previous studies [19,20], were included as confounders in the multivariable modified Poisson regressions to analyze the association between the exposures and depressive symptoms. Meanwhile, the generalized variance-inflation factor of covariates in the multivariable regressions were calculated to test for collinearity (Table S1 in Multimedia Appendix 1).

For subgroup analyses, all eligible participants were divided into different subgroups according to sex (male or female), age (<60 years old or ≥60 years old), type of community (village or city/town), solitude (living with a heterosexual partner or not), education (primary school and below or junior high school and above), alcohol consumption (yes or no), duration of sleep at night (<6 hours, 6‐8 hours, or ≥8 hours), nap after lunch (yes or no), physical activity (vigorous, moderate, mild, or none), and chronic conditions (≥2 chronic conditions or not). In the same way, multivariable modified Poisson regressions with the confounders, except the grouping variable, were performed to estimate the strength of association between the exposures and depressive symptoms.

There were missing values in variables for smoking habits, alcohol consumption, duration of sleep at night, nap after lunch, physical activity, social activity score, BADL score, and the number of body parts with pain, of which the proportions of missing values are shown in the Table S2 in Multimedia Appendix 1. Hot deck imputation was used to impute the missing values, and all statistical analyses were based on that data. Smoking habits, social activity score, BADL score, and the number of body parts with pain were not considered as confounders in the main analysis, due to the proportion of missing values being >40%. However, the above variables were adjusted for in the sensitivity analyses.

Multiple sensitivity analyses were conducted to validate the robustness of the association between the exposures and the outcome. First, the outcome of depressive symptoms was redefined as a CEDS-10 score of ≥11 or 12 in the multivariable modified Poisson regressions. Second, eligible participants diagnosed with emotional, nervous, or psychiatric problems in the fourth wave of the CHARLS were excluded. Third, to improve the comparability of the exposed groups with the control group, eligible participants aged >83 years in the non-WeChat users group were excluded since the oldest user in the WeChat users group was 83 years old (Table S3 in Multimedia Appendix 1). Fourth, additional confounders (smoking habits, BADL score, social activity score, and the number of body parts with pain) were adjusted for in the main analysis. Last, multivariable binary logistic regressions were performed to estimate the association between exposures and depressive symptoms.

All statistical analyses were completed by R 4.3.3 (R Core Team) with R packages *VIM*, *rqlm*, *car* and *forestploter* and the Mengte Cloud version 1.0 statistics platform (Wuxi Mengte Yi Shu Tong Co, Ltd). A 2-sided hypothesis test was used, and the significance level was set at *α*=.05.

### Ethical Considerations

The ethical approval for the CHARLS was approved by the Ethics Review Committee of Peking University (IRB00001052-11015). Prior to participation, all study participants were required to sign an informed consent form, thereby confirming their voluntary engagement in the survey process. The study data were anonymous. China’s Health Commission, Ministry of Science and Technology, and other departments jointly issued the Ethical Review Measures for Life Science and Medical Research involving Human Beings in February 2023, in which Article 32 mentioned 4 situations of exemption from ethical review. The secondary analysis of this public database CHARLS satisfied the first 2 of these: the data were free and publicly available and were available through a web-based application. Importantly, the data obtained from the platform were anonymous, and the identity information of all respondents was unknown. In addition, the study did not use biological samples from the survey subjects. Regarding compensation and informed consent, because this study was a secondary analysis, along with the subjects being anonymous, the respondents did not receive secondary compensation or sign a new informed consent form.

## Results

### The Characteristics of Eligible Participants

**Figure 1.**
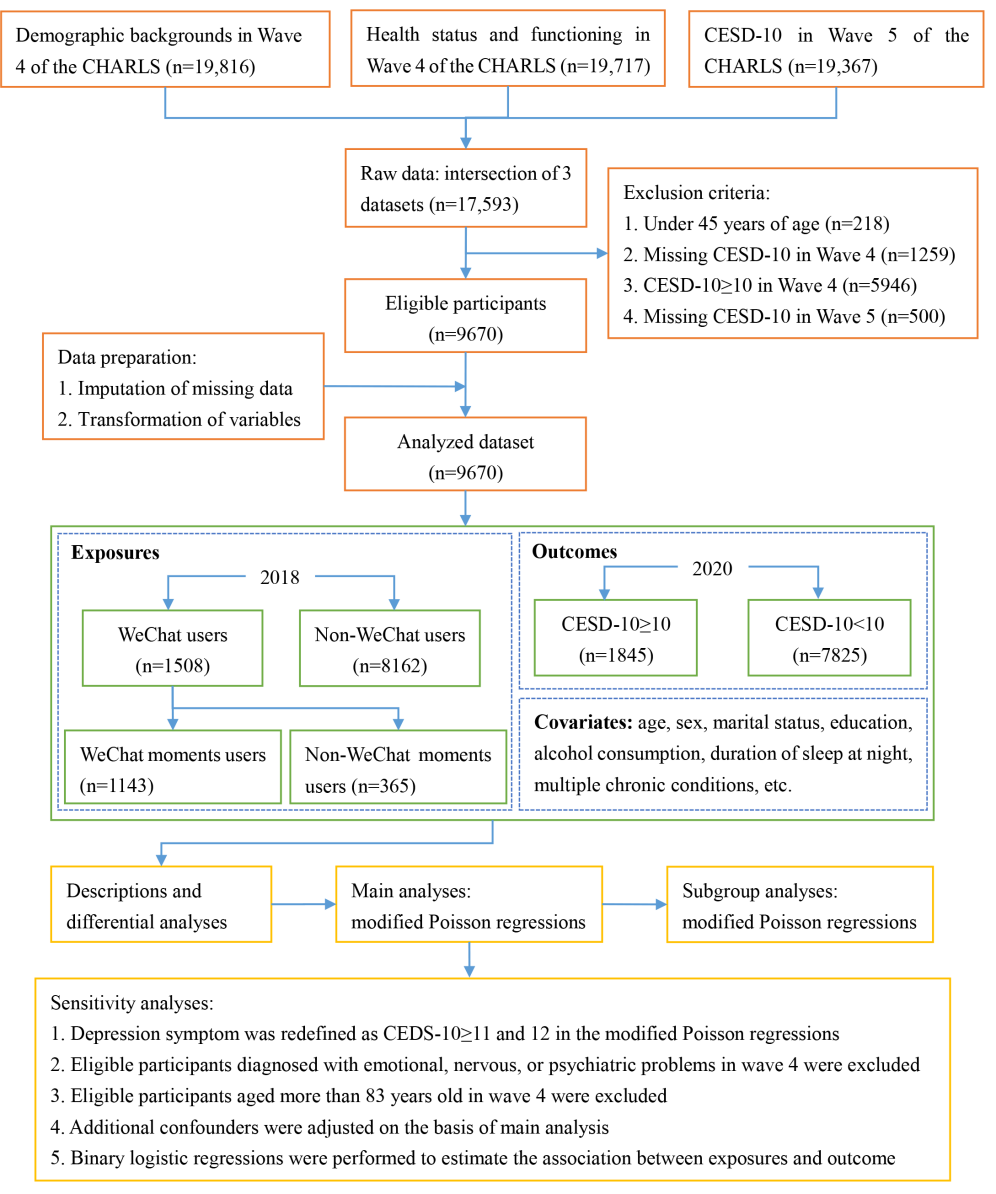
The flowchart for screening eligible participants, data governance, and statistical analysis. CESD-10: 10-item Center for Epidemiologic Studies Depression Scale; CHARLS: China Health and Retirement Longitudinal Study.

The process for screening participants is shown in Figure 1. A total of 9670 eligible participants were included in the final analysis. The mean age of these participants was 61.01 (SD 9.35) years. Of all eligible participants, 47.55% (n=4598) were female. A majority (n=6733, 69.63%) resided in villages, and 82.99% (n=8025) reported being married or cohabiting. Alcohol consumption was reported by 38.73% (n=3745) of participants, and 62.79% (n=6072) indicated they took a nap after lunch. A total of 92.84% (n=8978) engaged in physical activity, and 74.96% (n=7249) had been diagnosed with chronic conditions. The median scores of the CESD-10 were 4 (IQR 2-7) in the fourth wave and 6 (IQR 3-9) in the fifth wave. The incidence rate of depressive symptoms was 19.08% (95% CI 19.07%‐19.09%) from the fourth to fifth waves of the CHARLS (Table 1). Participants who used WeChat, regardless of whether they posted on WeChat moments, tended to be younger, male, non-religious, urban residents, or have a higher level of education. They were also more likely to be married or cohabiting, be current or former smokers, be alcohol consumers, sleep less than 8 hours at night, take afternoon naps, engage in physical activity, have high social activity scores, have low BADL scores, have fewer body parts in pain, and have no chronic conditions (Table 1 and S4 in Multimedia Appendix 1).

**Table 1.** The baseline characteristics and incidence of depressive symptoms of eligible participants in the cohort study based on the China Health and Retirement Longitudinal Study.

Characteristics	Total (n=9670)	Non-WeChat users (n=8162)	WeChat users (n=1508)		*P* value
			Non-WeChat moments users (n=365)	WeChat moments users (n=1143)	
Age (years), mean (SD)	61.01 (9.35)	62.05 (9.32)	55.68 (7.71)	55.28 (7.14)	<.001
Sex, n (%)					<.001
Male	5072 (52.45)	4204 (51.51)	204 (55.89)	664 (58.09)	
Female	4598 (47.55)	3958 (48.49)	161 (44.11)	479 (41.91)	
Race, n (%)					.48
Han	8990 (92.97)	7578 (92.84)	344 (94.25)	1068 (93.44)	
Minority	680 (7.03)	584 (7.16)	21 (5.75)	75 (6.56)	
Faith, n (%)					.02
Yes	946 (9.78)	821 (10.06)	40 (10.96)	85 (7.44)	
No	8724 (90.22)	7341 (89.94)	325 (89.04)	1058 (92.56)	
Type of community, n (%)					<.001
Village	6733 (69.63)	6085 (74.55)	176 (48.22)	472 (41.29)	
City/Town	2937 (30.37)	2077 (25.45)	189 (51.78)	671 (58.71)	
Education, n (%)					<.001
Illiterate	1721 (17.80)	1704 (20.88)	12 (3.29)	5 (0.44)	
Not finish primary school/home school/primary school	4040 (41.78)	3713 (45.49)	100 (27.40)	227 (19.86)	
Junior high school	2453 (25.37)	1903 (23.32)	133 (36.44)	417 (36.48)	
Senior high school and above	1456 (15.06)	842 (10.32)	120 (32.88)	494 (43.22)	
Marital status, n (%)					<.001
Married and cohabiting	8025 (82.99)	6727 (82.42)	307 (84.11)	991 (86.70)	
Married and separated	574 (5.94)	454 (5.56)	32 (8.77)	88 (7.70)	
Others	1071 (11.08)	981 (12.02)	26 (7.12)	64 (5.60)	
Smoking habits, n (%)					.006
Currently	333 (3.44)	271 (3.32)	8 (2.19)	54 (4.72)	
Ever	394 (4.07)	319 (3.91)	13 (3.56)	62 (5.42)	
Never	8943 (92.48)	7572 (92.77)	344 (94.25)	1027 (89.85)	
Alcohol consumption, n (%)					<.001
No	5925 (61.27)	5237 (64.16)	194 (53.15)	494 (43.22)	
Yes	3745 (38.73)	2925 (35.84)	171 (46.85)	649 (56.78)	
Duration of sleep at night, n (%)					<.001
<6 hours	2573 (26.61)	2229 (27.31)	88 (24.11)	256 (22.40)	
≥6 hours to <8 hours	4282 (44.28)	3456 (42.34)	190 (52.05)	636 (55.64)	
≥8 hours	2815 (29.11)	2477 (30.35)	87 (23.84)	251 (21.96)	
Nap after lunch, n (%)					<.001
No	3598 (37.21)	3129 (38.34)	131 (35.89)	338 (29.57)	
Yes	6072 (62.79)	5033 (61.66)	234 (64.11)	805 (70.43)	
Physical activity, n (%)					<.001
No	692 (7.16)	657 (8.05)	13 (3.56)	22 (1.92)	
Mild	2741 (28.35)	2321 (28.44)	96 (26.30)	324 (28.35)	
Moderate	3072 (31.77)	2462 (30.16)	143 (39.18)	467 (40.86)	
Vigorous	3165 (32.73)	2722 (33.35)	113 (30.96)	330 (28.87)	
Social activity score, median (IQR)	3 (2-5)	3 (2-4)	5 (3-6)	5 (3-8)	<.001
BADL^a^ score, mean (SD)	6.32 (1.02)	6.34 (1.04)	6.23 (0.71)	6.25 (0.90)	.006
Life satisfaction, n (%)					<.001
Completely/very	4010 (41.47)	3508 (42.98)	135 (36.99)	367 (32.11)	
Somewhat	5262 (54.42)	4319 (52.92)	211 (57.81)	732 (64.04)	
Not very/not at all	398 (4.12)	335 (4.10)	19 (5.21)	44 (3.85)	
Number of chronic conditions, n (%)					<.001
0	2421 (25.04)	1960 (24.01)	113 (30.96)	348 (30.45)	
1	2574 (26.62)	2179 (26.70)	98 (26.85)	297 (25.98)	
2	1971 (20.38)	1699 (20.82)	79 (21.64)	193 (16.89)	
3	1243 (12.85)	1051 (12.88)	45 (12.33)	147 (12.86)	
≥4	1461 (15.11)	1273 (15.60)	30 (8.22)	158 (13.82)	
Number of body parts with pain, median (IQR)	3 (2-6)	3 (2-6)	3 (1-5)	3 (1-5)	.007
CESD-10^b^ in 2020, median (IQR)	6 (3-9)	6 (3-10)	4 (2-7)	4 (1-7)	<.001
Depressive symptoms, n (%)					<.001
No	7825 (80.92)	6457 (79.11)	326 (89.32)	1042 (91.16)	
Yes	1845 (19.08)	1705 (20.89)	39 (10.68)	101 (8.84)	

a BADL: basic activities of daily living.

b CESD-10: 10-item Center for Epidemiologic Studies Depression Scale.

### Association Between Exposures and Depressive Symptoms

**Figure 2.**
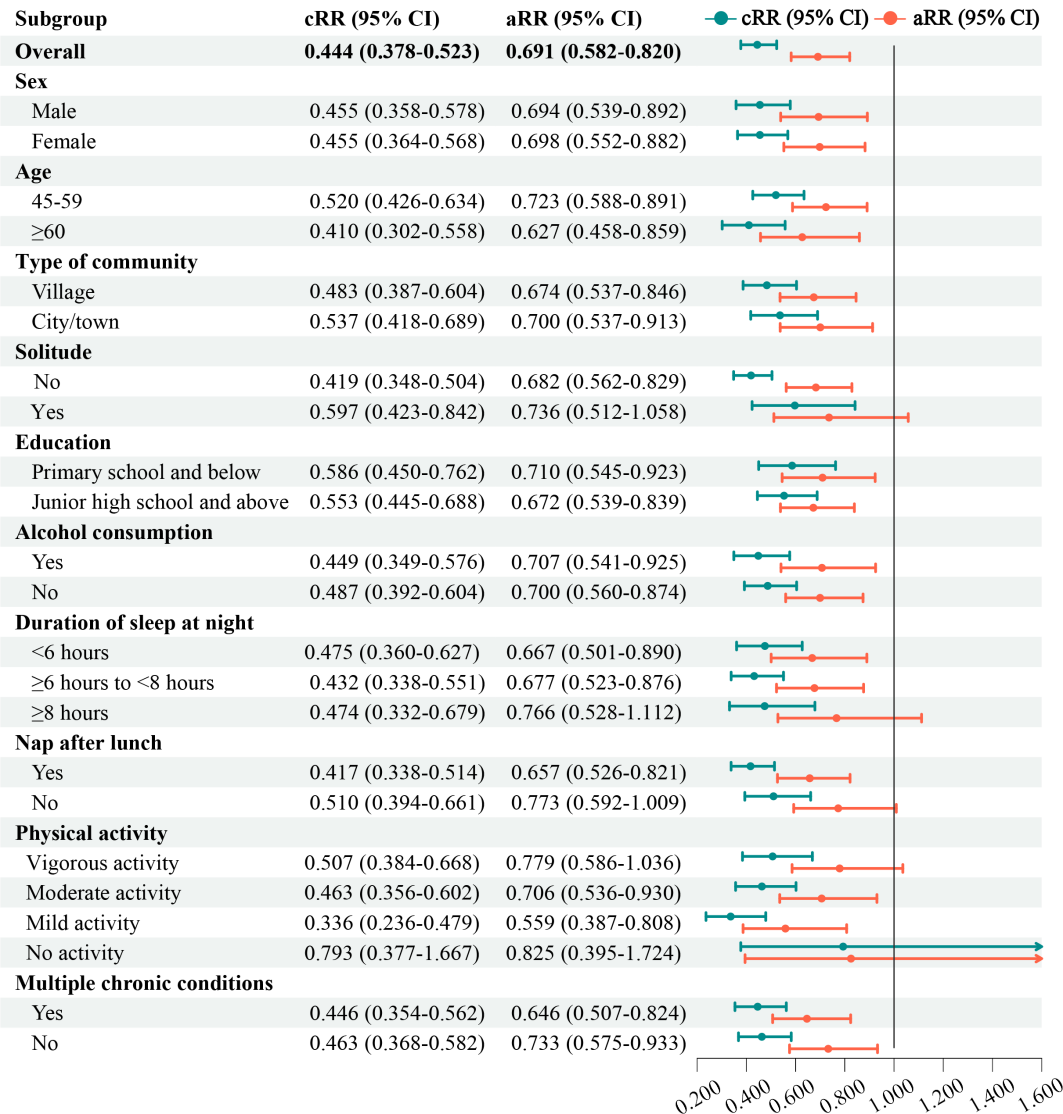
The association between using WeChat and risk of depressive symptoms among middle-aged and older Chinese adults in the cohort study based on the CHARLS. aRR: adjusted relative risk; cRR: crude relative risk.

As shown in Figure 2 and Table S5 in Multimedia Appendix 1, among all eligible participants, the risk of depressive symptoms in the WeChat users group was 0.556 times lower than that in the non-WeChat users group, as indicated by the crude relative risk (cRR) of 0.444 (95% CI 0.378‐0.523; *P*<.001) using a univariable modified Poisson regression. Furthermore, after adjusting for potential confounders in the multivariable modified Poisson regression models, the risk of depressive symptoms in the WeChat users group was 0.309 times lower than that in the non-WeChat users group (adjusted relative risk [aRR] 0.691, 95% CI 0.582‐0.820; *P*<.001). These results suggested a significant association between WeChat usage and a decreased risk of depressive symptoms, implying that WeChat may serve as a protective factor against depressive symptoms in community-based middle-aged and older Chinese adults.

Subgroup analyses showed that WeChat usage did not reduce the risk of depressive symptoms among those who were solitary, had a sleep duration of ≥8 hours at night, did not have nap after lunch, engaged in vigorous physical activity, or did not engage in any physical activity. However, in other subgroups, the result indicated that WeChat usage was associated with a lower risk of depressive symptoms.

**Figure 3.**
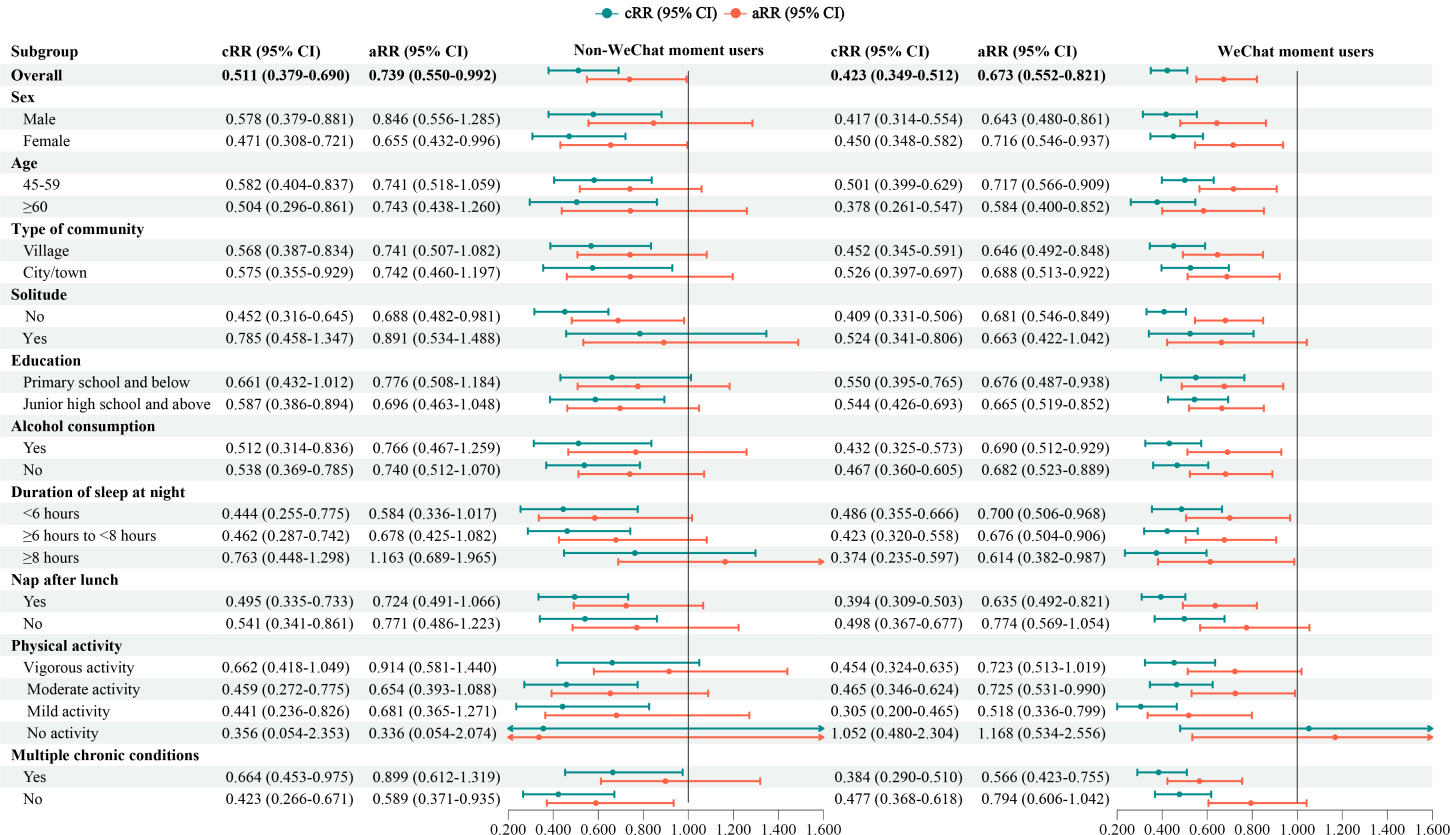
The association between using WeChat with or without posting WeChat moments and risk of depressive symptoms among middle-aged and older Chinese adults in the cohort study based on CHARLS. aRR: adjusted relative risk; cRR: crude relative risk.

As depicted in Figure 3 and Table S6 in Multimedia Appendix 1, compared to non-WeChat users, both the non-WeChat moments users (aRR 0.739, 95% CI 0.550‐0.992; *P*=.044) and WeChat moments users (aRR 0.673, 95% CI 0.552‐0.821; *P*<.001) exhibited a lower risk of depressive symptoms. In the subgroups of females (aRR 0.655, 95% CI 0.432‐0.996) and those without multiple chronic conditions (aRR 0.589, 95% CI 0.371–0.935), non-WeChat moments users had a lower risk of depressive symptoms compared to non-WeChat users. Additionally, with the exception of individuals who did not live with a spouse or opposite sex, did not nap after lunch, engaged in vigorous physical activity, did not engage in any physical activity, or did not have multiple chronic conditions, results from the subgroup analyses suggested that posting WeChat moments was associated with a reduced risk of depressive symptoms.

### Sensitivity Analyses

The results of sensitivity analyses are presented in Table 2. (1) When depressive symptoms were redefined as CESD-10 scores of ≥11 or 12, the multivariable Poisson regressions indicated that WeChat usage reduced the risk of depressive symptoms in middle-aged and older individuals, regardless of whether they posted WeChat moments. (2) The multivariable Poisson regression was performed again after excluding participants who had emotional, nervous, or psychiatric problems in the fourth wave of the CHARLS, and results were consistent with the main analysis. (3) Given that none of the WeChat users were over 83 years old, another multivariable Poisson regression was performed after excluding non-WeChat users over 83 years old, which yielded results similar to the main analysis. (4) Additional adjustments were made for smoking habits, social activity score, BADL score, and the number of body parts with pain. The outcome of depressive symptoms remained consistent with those of the main analysis. (5) Odds ratios from multivariable binary logistic regressions were used to estimate the associations, and the results demonstrated the robustness of the main analysis.

**Table 2.** Multiple sensitivity analyses to determine the association between exposures and depressive symptoms that were identified by the main analysis.

Sensitivity analysis and variables		β^a^ (SE)		aRR^b^/aOR^c^ (95% CI)	*P* value
Depressive symptoms was defined as CESD-10^d^ of ≥11 in the modified Poisson regressions					
Use WeChat (vs no)					
Yes		–0.370 (0.087)		0.691 (0.582‐0.820)	<.001
Use WeChat and post moments (vs do not use WeChat)					
Use WeChat and not post moments		–0.303 (0.151)		0.738 (0.549‐0.992)	.04
Use WeChat and post moments		–0.396 (0.101)		0.673 (0.552‐0.821)	<.001
Depressive symptoms was defined as CESD-10 of ≥12 in the modified Poisson regressions					
Use WeChat (vs no)					
Yes		–0.402 (0.100)		0.669 (0.550‐0.813)	<.001
Use WeChat and post moments (do not use WeChat)					
Use WeChat and not post moments		–0.267 (0.168)		0.766 (0.551‐1.064)	.11
Use WeChat and post moments		–0.457 (0.117)		0.633 (0.504‐0.796)	<.001
Participants diagnosed with emotional, nervous, or psychiatric problems in Wave 4 were excluded					
Use WeChat (vs no)					
Yes		–0.399 (0.089)		0.671 (0.564‐0.799)	<.001
Use WeChat and post moments (do not use WeChat)					
Use WeChat and not post moments		–0.325 (0.153)		0.723 (0.536‐0.975)	.03
Use WeChat and post moments		–0.428 (0.103)		0.652 (0.532‐0.799)	<.001
Participants aged more than 83 years in Wave 4 were excluded					
Use WeChat (vs no)					
Yes		–0.363 (0.088)		0.696 (0.586‐0.826)	<.001
Use WeChat and post moments (vs do not use WeChat)					
Use WeChat and not post moments		–0.298 (0.151)		0.742 (0.552‐0.998)	.048
Use WeChat and post moments		–0.388 (0.101)		0.678 (0.556‐0.827)	<.001
Smoking habits, social activity score, BADL^e^ score, and the number of body parts with pain were adjusted for the main analysis					
Use WeChat (vs no)					
Yes		–0.376 (0.090)		0.687 (0.576‐0.819)	<.001
** **Use WeChat and post moments (vs do not use WeChat)					
Use WeChat and not post moments		–0.306 (0.152)		0.736 (0.546‐0.992)	.045
Use WeChat and post moments		–0.403 (0.103)		0.668 (0.546‐0.818)	<.001
Multivariable logistic regressions for analyzing the association between exposures and depressive symptoms					
Use WeChat (vs no)					
Yes		–0.431 (0.102)		0.650 (0.532‐0.794)	<.001
Use WeChat and post moments (vs do not use WeChat)					
Use WeChat and not post moments		–0.361 (0.178)		0.697 (0.492‐0.988)	.04
Use WeChat and post moments		–0.457 (0.117)		0.633 (0.504‐0.797)	<.001

a β: regression coefficient.

b aRR: adjusted relative risk.

c aOR: adjusted odds ratio.

d CESD-10: 10-item Center for Epidemiologic Studies Depression Scale.

e BADL: basic activities of daily living.

## Discussion

### Principal Findings

This prospective national cohort study confirmed that using WeChat and posting WeChat moments was a protective factor for depressive symptoms among community based middle-aged and older adults in China. In terms of the absolute magnitude of the aRR, participants who post WeChat moments had a lower risk of depressive symptoms than those who do not post WeChat moments. The results of multiple sensitivity analysis also illustrated the robustness of the association between the exposures and depressive symptoms. In addition, the associations were heterogeneous across several subgroups. For example, irrespective of posting WeChat moments, the association between WeChat usage and the risk of depression was not significant among participants whose duration of sleep at night was more than or equal to 8 hours, while the association was statistically significant in the other two subgroups for the duration of sleep. Also, only in the subgroups where participants engaged in mild and moderate physical activity did using WeChat and posting WeChat moments mitigate the risk of depressive symptoms.

First, this study demonstrated that WeChat usage mitigated the risk of future depressive symptoms, which was consistent with the conclusions of 3 existing cross-sectional studies carried out by Qu et al [19], Wang et al [24], and Zhang and Liang [20]. Second, the study further suggested that posting WeChat moments was significantly associated with a decreased risk of depressive symptoms, and that was consistent with the finding of the cross-sectional study conducted by Zhang and Liang [20], but it was inconsistent with the finding of the cross-sectional study conducted by Qu et al [19]. The results from this prospective cohort study are more convincing because it meets a necessary and inarguable criterion for causal inference: the temporality of cause and effect [25]. Last, multiple subgroup analyses were performed to explore the heterogeneity of the associations for the different characteristics of the participants, which were not conducted in previous studies about WeChat usage and depression.

Many studies have shown that loneliness, social isolation, lack of family and social support, and lack of social activity are risk factors for anxiety and depression in adults [26-30]. On the one hand, WeChat is an instant message platform where WeChat users can communicate with family and friends to relieve the feeling of loneliness, especially for older adults whose children or relatives are not around them [31]. Importantly, a video call on WeChat allows users to see each other, which is more convenient for emotional expression and perception and users can obtain emotional support. WeChat users not only can maintain and improve existing social relationships through WeChat, but they can also expand their social circle to make more like-minded friends and engage in more social activity [32].

WeChat moments is another common and primary function of WeChat, which is an emotional space composed of words, pictures, and short videos posted by users. Users of WeChat moments can post what is happening around them or express their feelings by text or video, browse the content posted or reproduced by their WeChat friends, and interact with their friends through the act of liking and commenting on posts and short videos. Similarly, users can obtain emotional support and emotional communication, as well as release negative emotions by posting WeChat moments. Therefore, the results of Nan et al [33] were supported, which found that internet usage, including WeChat usage, could directly or indirectly reduce the risk of depression through improving interpersonal relationships. Since solitude, a longer sleep duration, and no physical activity are all risk factors for depressive symptoms, it is difficult to offset the negative impact of these factors by using WeChat or posting WeChat moments. Therefore, the heterogeneity of the associations among certain subgroups was observed. Importantly, the heterogeneity of the associations shown in the subgroup analysis suggested that a one-size-fits-all model should be used with caution, and recommendations for individualization or stratified treatment are necessary, especially when research is used to inform policy and clinicians’ decisions [34-36].

### Strengths and Limitations

There are several strengths of this study. First, this study is a prospective national cohort study, of which the sample sizes are large and the participants are representative. Second, modified Poisson regressions were used to precisely estimate the strength of the associations, which is a statistical approach more appropriate for this scenario given the high incidence of depressive symptoms. Third, multiple subgroups analyses were performed to explore the heterogeneity of the associations between the exposures and depressive symptoms among participants with different characteristics. Last, multiple sensitivity analyses were performed to validate the robustness of the associations between the exposures and depressive symptoms based on the main analysis.

Meanwhile, there are several limitations of this study. First, as the questions about WeChat usage and WeChat moments usage were only collected in the 2018 and 2020 surveys in the CHARLS database, the data for only these two surveys were used in this study. The corresponding follow-up period in this study was short, and a longer follow-up is needed to further confirm our conclusion. Second, the use of subgroups and more exposure groups (WeChat users were further divided into WeChat moments users and non-WeChat moments users) reduced the sample size and statistical power of the regression analyses. In particular, fewer participants used WeChat but did not post moments, which may have resulted in wide CIs for the associations and heterogeneity of the associations that are not easily explained. Third, some studies have suggested that excessive SMU or problematic SMU may increase the risk of depression [16,37,38]. However, the CHARLS database lacks relevant information on the frequency or duration of WeChat and WeChat moments usage, so this study cannot analyze the association between the frequency of WeChat use and depressive symptoms. Fourth, limited by the information collected in the database, other factors behind the use of WeChat and WeChat moments that may influence depressive symptoms were not considered in this study, such as the personality of participants (ie, outgoing or introverted). These factors can relate to whether participants post moments and affect the risk of depressive symptoms. Last, WeChat is only one of many social media software, and the participants may also use other social media software in addition to WeChat. Also, limited by the relevant information provided by CHARLS, this study was unable to investigate the effects of other social media and the joint use of them on mental health.

### Conclusion

In summary, the prevention of depression in community-based middle-aged and older adults calls for the joint efforts of individuals, families, and society. On the one hand, middle-aged and older adults are recommended to moderately use social media and the internet to learn more about new things or keep in touch with family and friends [39]. At the same time, middle-aged and older adults are recommended to participate in offline social activities and physical activity, if their physical capacity allows. On the other hand, it is suggested that children should actively contact their older family members, both electronically and offline, to provide them with emotional and material support. In addition, especially for older adults, it is recommended that the government and community organizations provide more community support [40], such as group recreational activities, which can also be important for the prevention of depression. In addition, the next waves of the CHARLS data will be obtained to determine the impact of using WeChat and posting WeChat moments on the long-term risk of depressive symptoms.

## Supplementary material

10.2196/62730 Multimedia Appendix 1 Additional data tables.

## Abbreviations

aRR: adjusted relative risk
BADL: basic activities of daily living
CESD-10: 10-item Center for Epidemiologic Studies Depression Scale
CHARLS: China Health and Retirement Longitudinal Study
cRR: crude relative risk
SMU: social media usage
