# Supplementary material for: The Association Between Posting WeChat Moments and the Risk of Depressive Symptoms Among Middle-Aged and Older Chinese Adults: Prospective National Cohort Study
Source: JMIR Public Health Surveill. 2025 Jan 13;11:e62730. doi: 10.2196/62730 (PMC11745077; doi:10.2196/62730)
Supplement: Multimedia Appendix 1 [file publichealth-v11-e62730-s001.doc]

**Multimedia Appendix 1**

**Supplemental Table 1.** Generalized variance-inflation factors of covariates in multivariable regressions

| Multivariable regressions | GVIF | Df | GVIF^(1/(2*Df)) |
| --- | --- | --- | --- |
| Association between WeChat usage and depressive symptoms | |  |  |
| WeChat usage | 1.160095 | 1 | 1.077077 |
| Age | 1.376043 | 1 | 1.173048 |
| Sex | 1.444985 | 1 | 1.202075 |
| Type of community | 1.179070 | 1 | 1.085850 |
| Education | 1.454207 | 3 | 1.064399 |
| Faith | 1.030628 | 1 | 1.015199 |
| Marital status | 1.161310 | 2 | 1.038095 |
| Alcohol consumption | 1.276280 | 1 | 1.129726 |
| Duration of sleep at night | 1.049813 | 2 | 1.012227 |
| Nap after lunch | 1.025065 | 1 | 1.012455 |
| Physical activity | 1.173469 | 3 | 1.027019 |
| Life satisfaction | 1.047256 | 2 | 1.011610 |
| Number of chronic conditions | 1.079918 | 4 | 1.009657 |
| Association between WeChat usage with or without posting WeChat moment and depressive symptoms | | | |
| WeChat and moments usage | 1.164115 | 2 | 1.038721 |
| Age | 1.375042 | 1 | 1.172622 |
| Sex | 1.441711 | 1 | 1.200713 |
| Type of community | 1.178751 | 1 | 1.085703 |
| Education | 1.447598 | 3 | 1.063591 |
| Faith | 1.157934 | 2 | 1.037340 |
| Marital status | 1.276223 | 1 | 1.129700 |
| Alcohol consumption | 1.049137 | 2 | 1.012064 |
| Duration of sleep at night | 1.025191 | 1 | 1.012517 |
| Nap after lunch | 1.171137 | 3 | 1.026679 |
| Physical activity | 1.047297 | 2 | 1.011620 |
| Life satisfaction | 1.078227 | 4 | 1.009459 |
| Number of chronic conditions |  |  |  |

GVIF: Generalized variance-inflation factors, Df : Degree of freedom

**Supplemental Table 2.** The variables with missing value and the proportion of missing values

| Variables | Count (n) | Proportion (%) |
| --- | --- | --- |
| Smoke habits | 3948 | 40.827 |
| Alcohol consumption | 1 | 0.010 |
| Duration of sleep at night | 1 | 0.010 |
| Nap after lunch | 1 | 0.010 |
| Physical activity | 1 | 0.010 |
| Social activity score | 4134 | 42.751 |
| BADL score | 3930 | 40.641 |
| The number of body parts with pain | 4638 | 47.963 |

BADL: basic activity of daily living

**Supplemental Table 3. The summary of age in each groups**

| Age, years | WeChat users | | |
| --- | --- | --- | --- |
|  | No | Yes, WeChat moments users | |
|  |  | No | Yes |
| Minimum value | 45.00 | 45.00 | 45.00 |
| First quantile | 54.00 | 50.00 | 50.00 |
| Median | 62.00 | 54.00 | 54.00 |
| Mean | 62.05 | 55.68 | 55.28 |
| Third quantile | 68.00 | 61.00 | 60.00 |
| Maximum value | 108.00 | 82.00 | 83.00 |

**Supplemental Table 4.** The characteristic of eligible participants by WeChat usage in the cohort study

| Characteristics | WeChat users | | *P* |
| --- | --- | --- | --- |
|  | Yes (*n*=1508) | No (*n*=8162) |  |
| Age, Mean±SD | 55.38±7.28 | 62.05±9.32 | <0.001 |
| **Sex, *n* (%)** |  |  | <0.001 |
| Male | 868 (57.56) | 4204 (51.51) |  |
| Female | 640 (42.44) | 3958 (48.49) |  |
| **Race, *n* (%)** |  |  | 0.271 |
| Han | 1412 (93.63) | 7578 (92.84) |  |
| Minority | 96 (6.37) | 584 (7.16) |  |
| **Faith, *n* (%)** |  |  | 0.034 |
| Yes | 125 (8.29) | 821 (10.06) |  |
| No | 1383 (91.71) | 7341 (89.94) |  |
| **Type of community, *n* (%)** |  |  | <0.001 |
| Village | 648 (42.97) | 6085 (74.55) |  |
| City/Town | 860 (57.03) | 2077 (25.45) |  |
| **Education, *n* (%)** |  |  | <0.001 |
| Illiteracy | 17 (1.13) | 1704 (20.88) |  |
| Not finish primary school/Home school/ Primary school | 327 (21.68) | 3713 (45.49) |  |
| Junior high school | 550 (36.47) | 1903 (23.32) |  |
| Senior high school and above | 614 (40.72) | 842 (10.32) |  |
| **Marital status, *n* (%)** |  |  | <0.001 |
| Married and cohabiting | 1300 (86.21) | 6730 (82.46) |  |
| Married and separated | 120 (7.96) | 454 (5.56) |  |
| Others | 88 (5.84) | 978 (11.98) |  |
| **Smoke habits, *n* (%)** |  |  | 0.042 |
| Current | 62 (4.11) | 271 (3.32) |  |
| Ever | 75 (4.97) | 319 (3.91) |  |
| Never | 1371 (90.92) | 7572 (92.77) |  |
| **Alcohol consumption, *n* (%)** |  |  | <0.001 |
| No | 688 (45.62) | 5237 (64.16) |  |
| Yes | 820 (54.38) | 2925 (35.84) |  |
| **Duration of sleep at night, *n* (%)** |  |  | <0.001 |
| < 6 hours | 344 (22.81) | 2229 (27.31) |  |
| ≥ 6 hours to < 8 hours | 826 (54.77) | 3456 (42.34) |  |
| ≥ 8 hours | 338 (22.41) | 2477 (30.35) |  |
| **Nap after lunch, *n* (%)** |  |  | <0.001 |
| No | 469 (31.10) | 3129 (38.34) |  |
| Yes | 1039 (68.90) | 5033 (61.66) |  |
| **Physical activity, *n* (%)** |  |  | <0.001 |
| No | 35 (2.32) | 657 (8.05) |  |
| Mild | 420 (27.85) | 2321 (28.44) |  |
| Moderate | 610 (40.45) | 2462 (30.16) |  |
| Vigorous | 443 (29.38) | 2722 (33.35) |  |
| Social activity score, M (IQR) | 5.00 (3.00, 7.00) | 3.00 (2.00, 4.00) | <0.001 |
| BADL score, Mean±SD | 6.25±0.86 | 6.34±1.04 | <0.001 |
| **Life satisfaction, *n* (%)** |  |  | <0.001 |
| Completely/very | 502 (33.29) | 3508 (42.98) |  |
| Somewhat | 943 (62.53) | 4319 (52.92) |  |
| Not very/not at all | 63 (4.18) | 335 (4.10) |  |
| **Number of chronic conditions, *n* (%)** |  |  |  |
| 0 | 523 (34.68) | 2402 (29.43) |  |
| 1 | 418 (27.72) | 2295 (28.12) |  |
| 2 | 276 (18.30) | 1572 (19.26) |  |
| 3 | 156 (10.34) | 920 (11.27) |  |
| ≥4 | 135 (8.95) | 973 (11.92) |  |
| Number of parts with pain, M (IQR) |  |  |  |
| CESD-10 in 2020, M (Q_1_, Q_3_) | 4.00 (2.00, 7.00) | 6.00 (3.00, 10.00) | <0.001 |
| **Depression symptoms, *n* (%)** |  |  | <0.001 |
| No | 1368 (90.72) | 6457 (79.11) |  |
| Yes | 140 (9.28) | 1705 (20.89) |  |

BADL: basic activity of daily living

**Supplemental Table 5.** The association between using WeChat and depressive symptom among all eligible participants after adjusting confounders

| Variables | coefficient | *RR* | *SE* | *LCI* | *UCI* | *P* |
| --- | --- | --- | --- | --- | --- | --- |
| (Intercept) | -2.296 | - | - | - | - | <.001 |
| **WeChat users** |  |  |  |  |  |  |
| No | Reference |  |  |  |  |  |
| Yes | -0.370 | 0.691 | 0.087 | 0.5820 | 0.820 | <.001 |
| Age | 0.009 | 1.009 | 0.003 | 1.0043 | 1.014 | <.001 |
| **Sex** |  |  |  |  |  |  |
| Male | Reference |  |  |  |  |  |
| Female | 0.267 | 1.307 | 0.050 | 1.185 | 1.441 | <.001 |
| **Type of community** |  |  |  |  |  |  |
| Village | Reference |  |  |  |  |  |
| City/Town | -0.365 | 0.694 | 0.056 | 0.622 | 0.775 | <.001 |
| **Education** |  |  |  |  |  |  |
| Illiteracy | Reference |  |  |  |  |  |
| Not finish primary school/  Home school/ Primary school | -0.078 | 0.925 | 0.052 | 0.836 | 1.024 | 0.133 |
| Junior high school | -0.332 | 0.718 | 0.071 | 0.625 | 0.824 | <.001 |
| Senior high school and above | -0.596 | 0.551 | 0.099 | 0.454 | 0.670 | <.001 |
| **Marital status** |  |  |  |  |  |  |
| Married and cohabiting | Reference |  |  |  |  |  |
| Married and separated | 0.192 | 1.211 | 0.082 | 1.031 | 1.423 | 0.020 |
| Others | 0.007 | 1.007 | 0.063 | 0.889 | 1.140 | 0.918 |
| **Faith** |  |  |  |  |  |  |
| Yes | Reference |  |  |  |  |  |
| No | -0.003 | 0.997 | 0.064 | 0.879 | 1.132 | 0.967 |
| **Alcohol consumption** |  |  |  |  |  |  |
| No | Reference |  |  |  |  |  |
| Yes | -0.091 | 0.913 | 0.051 | 0.827 | 1.008 | 0.072 |
| **Duration of sleep at night** |  |  |  |  |  |  |
| < 6 hours |  |  |  |  |  |  |
| ≥ 6 hours to < 8 hours | -0.292 | 0.747 | 0.048 | 0.680 | 0.820 | <.001 |
| ≥ 8 hours | -0.330 | 0.719 | 0.053 | 0.648 | 0.798 | <.001 |
| **Nap after lunch** |  |  |  |  |  |  |
| No | Reference |  |  |  |  |  |
| Yes | -0.063 | 0.939 | 0.041 | 0.866 | 1.019 | .130 |
| **Physical activity** |  |  |  |  |  |  |
| No | Reference |  |  |  |  |  |
| Mild | 0.104 | 1.110 | 0.082 | 0.944 | 1.305 | .206 |
| Moderate | 0.054 | 1.056 | 0.083 | 0.898 | 1.241 | .511 |
| Vigorous | 0.142 | 1.152 | 0.081 | 0.983 | 1.350 | .080 |
| **Life satisfaction** |  |  |  |  |  |  |
| Completely/very | Reference |  |  |  |  |  |
| Somewhat | 0.201 | 1.222 | 0.044 | 1.122 | 1.332 | <.001 |
| Not very/not at all | 0.666 | 1.946 | 0.075 | 1.681 | 2.252 | <.001 |
| **Number of chronic conditions** |  |  |  |  |  |  |
| 0 | Reference |  |  |  |  |  |
| 1 | 0.162 | 1.176 | 0.062 | 1.042 | 1.328 | .009 |
| 2 | 0.341 | 1.406 | 0.064 | 1.240 | 1.593 | <.001 |
| 3 | 0.458 | 1.580 | 0.071 | 1.375 | 1.816 | <.001 |
| ≥4 | 0.621 | 1.860 | 0.066 | 1.635 | 2.117 | <.001 |

RR, relative risk; SE, standard error; LCI, the lower limit of 95% confidence interval; UCI, the upper limit of 95% confidence interval

**Supplemental Table 6.** The association between posting WeChat moments and depressive symptom among all eligible participants after adjusting confounders

| Variables | coefficient | *RR* | *SE* | *LCI* | *UCI* | *P* |
| --- | --- | --- | --- | --- | --- | --- |
| (Intercept) | -2.2973 |  |  |  |  | <.001 |
| **WeChat moments users** | Reference |  |  |  |  |  |
| Non-WeChat users |  |  |  |  |  |  |
| No | -0.303 | 0.738 | 0.151 | 0.549 | 0.992 | .044 |
| Yes | -0.396 | 0.673 | 0.101 | 0.552 | 0.821 | <.001 |
| Age | 0.009 | 1.009 | 0.003 | 1.004 | 1.014 | <.001 |
| **Sex** |  |  |  |  |  |  |
| Male | Reference |  |  |  |  |  |
| Female | 0.268 | 1.307 | 0.050 | 1.185 | 1.441 | <.001 |
| **Type of community** |  |  |  |  |  |  |
| Village | Reference |  |  |  |  |  |
| City/Town | -0.364 | 0.695 | 0.056 | 0.622 | 0.776 | <.001 |
| **Education** |  |  |  |  |  |  |
| Illiteracy | Reference |  |  |  |  |  |
| Not finish primary school/Home school/ Primary school | -0.078 | 0.925 | 0.052 | 0.836 | 1.024 | .132 |
| Junior high school | -0.331 | 0.718 | 0.071 | 0.625 | 0.824 | <.001 |
| Senior high school and above | -0.594 | 0.552 | 0.099 | 0.455 | 0.671 | <.001 |
| **Marital status** |  |  |  |  |  |  |
| Married and cohabiting | Reference |  |  |  |  |  |
| Married and separated | 0.191 | 1.211 | 0.082 | 1.031 | 1.422 | .020 |
| Others | 0.006 | 1.006 | 0.063 | 0.889 | 1.139 | .922 |
| **Faith** |  |  |  |  |  |  |
| Yes | Reference |  |  |  |  |  |
| No | -0.002 | 0.998 | 0.065 | 0.880 | 1.133 | .976 |
| **Alcohol consumption** |  |  |  |  |  |  |
| No | Reference |  |  |  |  |  |
| Yes | -0.091 | 0.914 | 0.051 | 0.827 | 1.009 | .074 |
| **Duration of sleep at night** |  |  |  |  |  |  |
| < 6 hours | Reference |  |  |  |  |  |
| ≥ 6 hours to < 8 hours | -0.292 | 0.747 | 0.048 | 0.680 | 0.821 | <.001 |
| ≥ 8 hours | -0.330 | 0.719 | 0.053 | 0.648 | 0.798 | <.001 |
| **Nap after lunch** |  |  |  |  |  |  |
| No | Reference |  |  |  |  |  |
| Yes | -0.062 | 0.940 | 0.041 | 0.866 | 1.019 | .132 |
| **Physical activity** |  |  |  |  |  |  |
| No | Reference |  |  |  |  |  |
| Mild | 0.105 | 1.110 | 0.082 | 0.945 | 1.305 | .204 |
| Moderate | 0.055 | 1.056 | 0.083 | 0.898 | 1.242 | .509 |
| Vigorous | 0.142 | 1.152 | 0.081 | 0.983 | 1.350 | .080 |
| **Life satisfaction** |  |  |  |  |  |  |
| Completely/very | Reference |  |  |  |  |  |
| Somewhat | 0.2008 | 1.2224 | 0.0437 | 1.1220 | 1.3319 | <.001 |
| Not very/not at all | 0.6655 | 1.9455 | 0.0746 | 1.6808 | 2.2521 | <.001 |
| **Number of chronic conditions** |  |  |  |  |  |  |
| 0 | Reference |  |  |  |  |  |
| 1 | 0.162 | 1.176 | 0.062 | 1.041 | 1.328 | .009 |
| 2 | 0.341 | 1.406 | 0.064 | 1.240 | 1.593 | <.001 |
| 3 | 0.458 | 1.581 | 0.071 | 1.376 | 1.816 | <.001 |
| ≥4 | 0.621 | 1.861 | 0.066 | 1.635 | 2.118 | <.001 |

RR, relative risk; SE, standard error; LCI, the lower limit of 95% confidence interval; UCI, the upper limit of 95% confidence interval
